# Supplementary material for: Learning curve and surgical outcome of robotic assisted colorectal surgery with ERAS program
Source: Sci Rep. 2022 Nov 29;12:20566. doi: 10.1038/s41598-022-24665-w (PMC9709162; doi:10.1038/s41598-022-24665-w)
Supplement: Supplementary file 1 — Supplementary Information 1. [file 41598_2022_24665_MOESM1_ESM.zip › LSC Raw data-Ñ[▒K/107/N_ñw╝╨░O▒Kñσ.pdf]

W52 / W52

臺中榮民總醫院
手術室護理記錄

[redacted]

基本資料

- 室別： 開刀房-06室，手術科別： CRS，手術類別： 常規，日期： 2020/12/04
- 進入時間： 2015 ； 麻醉開始時間： 2020 ； Time Out時間： 2140 ； 劃刀時間： 2141 ； 手術結束時間： 0015 送出時間： 0030 ；
- 麻醉方式： GE

  診斷： S-colon Ca with bleeding

  手術名稱： Laparoscopic Anterior resection and anastomosis - malignant

  手術局部用藥： NIL

  術前預防性抗生素：
  - 抗生素名稱一： Veterin 1GM IVD由麻醉護理人員給予
  - 時間： 2135
  - 上次簽章: 張湘玲

  ，追加預防性抗生素：
- 手術醫師： 蔣鋒帆，江世偉，陳映菁，陳凱祥
- 麻醉醫師： VS 謝昀叡
-
  - 刷手護士A ： 蘇敬涵，時間： 2015，接替刷手一： 張秀敏，時間： 2230，接替刷手二： 王明盈，時間： 2300
  - 流動護士A ： 林怡廷，時間： 2015，接替流動一： 張湘玲，時間： 2100，接替流動二： 邱櫂媄，時間： 2200
- 更多接替及流動護士： 接替流動三： 吳思薇，時間： 2300

皮膚狀況

A-V Shunt： 無

術前： 完整

記錄者： 上次簽章: 張湘玲

術後： 不完整

部位(術後)：

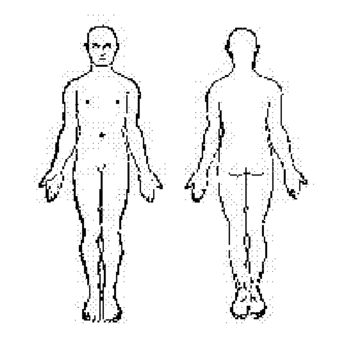

原術前雙眼紅腫、左內眼頭眼瞼外翻存
右肘皮膚約4cm*4cm壓紅可白存
薦部皮膚約8cm*2cm壓紅不可白存
上下唇氣管內管造成破皮及瘀血存
左鼻皮膚鼻胃管造成點狀壓紅存

記錄者： 吳思薇

手術準備

手術臥式 單一手術臥式

-
  - 手術臥式： 截石臥式
  - 使用附件： 溫毯 38 ℃，頭圈，腳架
  - 皮膚準備： 10%Aq Betadine，Hibiscrub，2% Chlorhexidine

- ○ 電燒： 使用電燒: 電極片位置： 其它： 迴路電極墊，記錄者： 上次簽章: 張湘玲

止血帶T： 無

植入物

- 植入物名稱/號碼/數量/部位： Ethicon endo GIA：60mm*4.1mm/1/腸斷端
  Ethcon 29mm EEA/腸吻合處

, 記錄者： 上次簽章: 吳思薇

標本送檢

- 細菌培養 無
- 細胞學 無
- 冰凍切片 無
- 病理標本
  組織名稱一： S colon
  ,
  組織名稱二： distal cut end
  ,
  組織名稱三： IMA root
- 截肢 無

標本交付 標本： 3 袋 ， 標本： 0 盒 ， 標本： 0 瓶 ， 細胞學： 0 管 ， Culture Tube： 0 管 ， 被交付醫師： CR 陳映菁 ，

- 記錄者： 吳思薇
- 時間： 0030

傷口術後敷料

傷口敷料：　　　傷口敷料： 無

- 請於圖上標示部位：

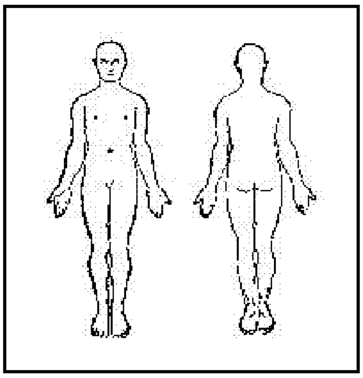

- 傷口敷料： mepore

導管

導管	天數	導管資訊	插入日期	插入時間	更換日期	拔除日期	拔除時間	拔除原因	預計拔除/更換	預計拔除/更換日期
Endotracheal Tube(1)	2	號碼:7.0 mm.; 部位:氣管; 註記:fix 22 cm;	2020/12/04	2120						
Foley(1)	2	號碼:16 Fr.; 部位:膀胱;	2020/12/04	2000						
Penrose Drain(1)	2	號碼:12 mm.; 部位:骨盆腔;	2020/12/04	2325						
Epidural PCA(1)	2	部位:硬脊膜;	2020/12/04	2000						
A-line(1)	2	號碼:20 ; 部位:左手;	2020/12/04	2130						
Sump NG Tube(1)	2	號碼:16 Fr.; 部位:胃;	2020/12/04	2000						

用物計數

- 手術中出血量： minimal c.c.
- 手術中尿量： 200 c.c.
-
  - ○ 其它： 無

用物計數： 多切口同時縫合

- 1.器械： 正確，2.縫針： 正確，3.刀片： 正確，4.紗布： 正確，5.腹部墊： 未使用，6.紗球： 未使用，7.PEANUTS ： 未使用，8.紗條： 未使用，9.絲帶： 未使用，10.針頭： 未使用，11.Vessel loop： 未使用，12.Boots： 未使用，13.Jelco： 未使用，14.棉條： 未使用，
    - 第一次：
        - 刷手護士 ： 王明盈
        - 流動護士： 上次簽章: 吳思薇
        - 時間： 2340

    ,
    - 第二次：
        - 刷手護士 ： 王明盈
        - 流動護士： 上次簽章: 吳思薇
        - 時間： 2345

    ,
    - 第三次：
        - 刷手護士 ： 王明盈
        - 流動護士： 上次簽章: 吳思薇
        - 時間： 2350

備註

備註：NIL

- 記錄者： 上次簽章: 吳思薇
- 時間： 0005

填寫護理人員 吳思薇

正式摘要

W52 / W52

doctorRank:B
